# Nutritional virulence of *Francisella tularensis*

**DOI:** 10.3389/fcimb.2013.00112

**Published:** 2013-12-31

**Authors:** Marina Santic, Yousef Abu Kwaik

**Affiliations:** ^1^Department of Microbiology and Parasitology, University of RijekaRijeka, Croatia; ^2^Department of Microbiology and Immunology, College of Medicine, University of LouisvilleLouisville, KY, USA

**Keywords:** tularemia, legionnaires disease, legionella, anaplasma phagocytophilum, autophagy

One of the most fundamental aspects of infectious diseases is microbial acquisition of nutrients *in vivo*, which impacts virulence and antibiotic treatment. Therefore, it is not surprising that part of the innate host defense against microbial infection is to limit access of various invading pathogens to sources of host nutrients (Abu Kwaik and Bumann, 2013; Eisenreich et al., 2013). Despite this host nutritional restriction, there has been a long held presumption that the host cell cytosol is a rich haven of plentiful nutrients sufficient to power proliferation of various intracellular pathogens. However, recent studies on the two intra-vacuolar pathogens *Anaplasma phagocytophilum* (Niu et al., 2012), *Legionella pneumophila* (Price et al., 2011) and the cytosolic pathogen *Francisella tularensis* (Steele et al., 2013) have clearly shown that the levels of amino acids in the host cell cytosol are not sufficient to meet the tremendous demands for carbon, energy, and nitrogen to power the robust intracellular proliferation of these pathogens (Abu Kwaik and Bumann, 2013). Therefore, these intracellular pathogens have evolved with efficient strategies to boost the levels of host amino acids to meet their demands for high levels of carbon and energy sources (Abu Kwaik and Bumann, 2013). There is an emerging paradigm of specific microbial strategies that directly trigger the host cell to boost the cellular levels of essential microbial nutrients, and this paradigm has been designated as nutritional virulence strategies (Abu Kwaik and Bumann, 2013). This opinion article is focused on nutritional virulence of *F. tularensis*.

Upon entry into mammalian and arthropod-derived cells, the vacuole harboring *F. tularensis* matures into an acidified late endosome-like phagosome followed by rapid bacterial egress into the cytosol within 30 min of bacterial entry (Akimana and Kwaik, 2011; Asare and Abu Kwaik, 2011). Amino acids are major sources of carbon and energy for *F. tularensis* but the levels of amino acids in the host cell cytosol are not sufficient to meet the pathogen metabolic needs for the robust intracellular proliferation. Although the mammalian cell cytosol is presumed to have plentiful levels of amino acids, the cells are auxotrophic for His, Lys, Met, Phe, Trp, Leu, Ile,Val, and Thr, while Cys is considered semi-essential and is the most limiting amino acid in mammals (Price et al., 2013). *F. tularensis* is auxotrophic for six amino acids that include His, placeLys, Met, Cys, Arg, and Tyr. Therefore, mammalian cells have limited capacity to provide 4 of the 6 amino acids essential for *F. tularensis*, and this synchronization in amino acid auxotrophy could have played a factor in nutritional and metabolic evolution as well as adaptation of *F. tularensis* to the intracellular environment of mammalian cells (Price et al., 2013). Similar to *L. pneumophila, F. tularensis* is auxotrophic for Cys and requires high levels of this amino acids, which is metabolized into pyruvate that feeds the TCA cycle as the major metabolic pathway for generation of energy and some amino acids. To boost the cellular levels of Cys in the host cell cytosol, *F. tularensis* exploits host glutathione (GSH), which is a non-ribosomal tri-peptide (L-γ-L-glutamyl-L-Cysteinyl-glycine) and is the most abundant source of Cys in the host cytosol (Alkhuder et al., 2009; Meibom and Charbit, 2010). The γ-glutamyl transpeptidase (Ggt) enzyme of *F. tularensis* cleaves GSH to liberate Cys (Alkhuder et al., 2009; Meibom and Charbit, 2010). The *ggt* mutant of *F. tularensis* exhibits a severe intracellular growth defect but is rescued upon supplementation of Cys (Alkhuder et al., 2009).

In addition to requirements to boost cellular levels of Cys, *F. tularensis* signals the host cell to boost levels of all amino acids by triggering the host macroautophagy degradation machinery, which is required for optimal intracellular bacterial growth (Steele et al., 2013). Inhibition of the host macroautophagy diminishes intracellular growth of *F. tularensis*, but the defect is rescued upon supplementation of excess amino acids or pyruvate (Steele et al., 2013). The macroautophagy degradation pathway triggered by *F. tularensis* is non-canonical, since it is independent of the ATG5 autophagy pathway. Thus, in addition to degradation of the host GSH, an additional nutritional virulence strategy for *F. tularensis* is to trigger a major host degradation machinery to boost the cellular levels of amino acids, and both nutritional virulence strategies are required for intracellular proliferation and manifestation of tularemia.

Availability of excess amino acids in the host cell cytosol is of no benefit to the pathogen if they are not imported by efficient bacterial transporters. The *Francisella* genome encodes numerous amino acids transporters and predicted secondary carriers that include the major facilitator superfamily (MFS), such as several putative orthologous of PhtA, which import threonine (Meibom and Charbit, 2010). A novel amino acid transporter, designated AnsP, belongs to the MFS secondary transporters has been recently characterized in *F. tularensis* (Gesbert et al., 2013). The AnsP MFS transporter imports Asn, which is a non-essential amino acid for *F. tularensis* or mammalian cells. The AnsP transporter is not required for growth of *F. tularensis in vitro* or for bacterial egress from the vacuole into the cytosol of mammalian macrophages. However, AnsP is essential for proliferation of *F. tularensis* in mammalian macrophages and for manifestation of tularemia in the mouse model (Gesbert et al., 2013). Defect of the *ansP* mutant in intracellular growth is overcome upon supplementation of the cells with Asn or Asn-containing dipeptides, or by a combination of Asp and ammonium (Gesbert et al., 2013). In addition, the *ansP* mutant of *F. tularensis* is attenuated in the mouse model of tularemia but the attenuation is reversed upon injection of Asn. Thus, the non-essential amino acid Asn is important for intracellular proliferation of *F. tularensis* in the macrophage cytosol. It is most likely that there are other amino acids transporters in *F. tularensis* (Gesbert et al., 2013) and many of them would be required to import the respective amino acids needed for intracellular proliferation of *F. tularensis*. Therefore, in addition to microbial nutritional virulence strategies, microbial transporters of nutrients are potential targets for therapy against tularemia (Abu Kwaik, 2013).

It is possible that some intra-vacuolar pathogens may not be able to obtain sufficient amino acids from the host cell due to inefficient import of amino acids across the pathogen-containing vacuolar membrane. Therefore, triggering the host cell to elevate the cellular levels of amino acids would enhance their import across the pathogen-containing vacuolar membrane. However, it is very clear from the studies on *F. tularensis* that resides and proliferate in the host cell cytosol that it requires triggering host autophagic degradation as a source of amino acids. This clearly documents that the host cell cytosol does not have sufficient levels of amino acids to support intracellular proliferation of many intra-vacuolar and cytosolic pathogens. The need to raise the cellular levels of amino acids by some pathogens is likely more crucial for intracellular pathogens with shorter generation times in the host cell that does not have sufficient levels to meet the high metabolic needs of the pathogen for additional sources of carbon and energy. The intra-vacuolar *L. pneumophila* (Price et al., 2011) and the cytosolic *F. tularensis* (Alkhuder et al., 2009; Meibom and Charbit, 2010; Steele et al., 2013) are classic examples for such group of pathogens. It is most likely that many other intracellular pathogens trigger various host degradation machinery, intercept host biosynthetic pathways, or other essential sources of carbon and energy as major sources of amino acids needed for robust build up of bacterial biomass during intracellular replication.

## References

[B1] Abu Kwaik Y. (2013). Targeting nutrient retrieval by *Francisella tularensis*. Front. Cell. Infect. Microbiol. 3:64 10.3389/fcimb.2013.00064 24133657 PMC3796267

[B2] Abu Kwaik Y. Bumann D. (2013). Microbial quest for food *in vivo*: ‘nutritional virulence’ as an emerging paradigm. Cell Microbiol. 15, 882–890 10.1111/cmi.12138 23490329

[B3] Akimana C. Kwaik Y. A. (2011). Francisella-arthropod vector interaction and its role in patho-adaptation to infect mammals. Front. Microbiol. 2:34 10.3389/fmicb.2011.00034 21687425 PMC3109307

[B4] Alkhuder K. Meibom K. L. Dubail I. Dupuis M. Charbit A. (2009). Glutathione provides a source of cysteine essential for intracellular multiplication of *Francisella tularensis*. PLoS Pathog. 5:e1000284 10.1371/journal.ppat.1000284 19158962 PMC2629122

[B5] Asare R. Kwaik Y. A. (2011). Exlploitation of the host cell biology and evasion of immunity by *Francisella tularensis*. Front. Microbiol. 1:145 10.3389/fmicb.2010.00145 21687747 PMC3109322

[B6] Eisenreich W. Heesemann J. Rudel T. Goebel W. (2013). Metabolic host responses to infection by intracellular bacterial pathogens. Front. Cell. Infect. Microbiol. 3:24 10.3389/fcimb.2013.00024 23847769 PMC3705551

[B7] Gesbert G. Ramond E. Rigard M. Frapy E. Dupuis M. Dubail I. (2013). Asparagine assimilation is critical for intracellular replication and dissemination of Francisella. Cell Microbiol. [Epub ahead of print]. 10.1111/cmi.12227 24134488

[B8] Meibom K. L. Charbit A. (2010). *Francisella tularensis* metabolism and its relation to virulence. Front. Microbiol. 1:140 10.3389/fmicb.2010.00140 21687763 PMC3109416

[B9] Niu H. Xiong Q. Yamamoto A. Hayashi-Nishino M. Rikihisa Y. (2012). Autophagosomes induced by a bacterial Beclin 1 binding protein facilitate obligatory intracellular infection. Proc. Natl. Acad. Sci. U.S.A. 109, 20800–20807 10.1073/pnas.1218674109 23197835 PMC3529060

[B10] Price C. T. Al-Quadan T. Santic M. Rosenshine I. Abu Kwaik Y. (2011). Host proteasomal degradation generates amino acids essential for intracellular bacterial growth. Science 334, 1553–1557 10.1126/science.1212868 22096100

[B11] Price C. T. D. Richards A. M. Von Dwingelo J. E. Samara H. A. (2013). Legionella pneumophila synchronization of amino acid auxotrophy and its role in adaptation and pathogenic evolution to the amoeba host. Environ. Microbiol. 4, 307–314 10.1111/1462-2920.12290 24112119 PMC3946891

[B12] Steele S. Brunton J. Ziehr B. Taft-Benz S. Moorman N. Kawula T. (2013). *Francisella tularensis* harvests nutrients derived via ATG5-independent autophagy to support intracellular growth. PLoS Pathog. 9:e1003562 10.1371/journal.ppat.1003562 23966861 PMC3744417

